# Transient Anomalous Diffusion MRI in Excised Mouse Spinal Cord: Comparison Among Different Diffusion Metrics and Validation With Histology

**DOI:** 10.3389/fnins.2021.797642

**Published:** 2022-02-15

**Authors:** Alessandra Caporale, Giovanni Battista Bonomo, Giulio Tani Raffaelli, Ada Maria Tata, Bice Avallone, Felix Werner Wehrli, Silvia Capuani

**Affiliations:** ^1^NMR and Medical Physics Laboratory, Institute for Complex Systems of National Research Council (CNR-ISC), Rome, Italy; ^2^Laboratory for Structural, Physiologic and Functional Imaging, Department of Radiology, University of Pennsylvania, Philadelphia, PA, United States; ^3^Department of Physics, Sapienza University of Rome, Rome, Italy; ^4^Department of Biology and Biotechnologies Charles Darwin, Sapienza University of Rome, Rome, Italy; ^5^Research Center of Neurobiology Daniel Bovet, Rome, Italy; ^6^Department of Biology, University of Naples Federico II, Naples, Italy; ^7^Centro Fermi, Museo Storico della Fisica e Centro Studi e Ricerche Enrico Fermi, Rome, Italy

**Keywords:** transient anomalous diffusion, q-space imaging, DTI, histology, diffusion parameters validation, mouse spinal cord, white matter (WM), microstructures

## Abstract

Neural tissue is a hierarchical multiscale system with intracellular and extracellular diffusion compartments at different length scales. The normal diffusion of bulk water in tissues is not able to detect the specific features of a complex system, providing nonlocal, diffusion measurement averaged on a 10-20 μm length scale. Being able to probe tissues with sub-micrometric diffusion length and quantify new local parameters, transient anomalous diffusion (tAD) would dramatically increase the diagnostic potential of diffusion MRI (DMRI) in detecting collective and sub-micro architectural changes of human tissues due to pathological damage. In DMRI, the use of tAD parameters quantified using specific DMRI acquisition protocols and their interpretation has often aroused skepticism. Although the derived formulas may accurately fit experimental diffusion-weighted data, the relationships between the postulated dynamical feature and the underlying geometrical structure remains elusive, or at most only suggestive. This work aimed to elucidate and validate the image contrast and information that can be obtained using the tAD model in white matter (WM) through a direct comparison between different diffusion metrics and histology. Towards this goal, we compared tAD metrics extracted from pure subdiffusion (α-imaging) and super-pseudodiffusion (γ-imaging) in excised mouse spinal cord WM, together with T2 and T2* relaxometry, conventional (normal diffusion-based) diffusion tensor imaging (DTI) and q-space imaging (QSI), with morphologic measures obtained by optical microscopy, to determine which structural and topological characteristics of myelinated axons influenced tAD contrast. Axon diameter (AxDiam), the standard deviation of diameters (SD_*ax.diam*_), axonal density (AxDens) and effective local density (ELD) were extracted from optical images in several WM tracts. Among all the diffusion parameters obtained at 9.4 T, γ-metrics confirmed a strong dependence on magnetic in-homogeneities quantified by R2* = 1/T2* and showed the strongest associations with AxDiam and ELD. On the other hand, α-metrics showed strong associations with SD_*ax.diam*_ and was significantly related to AxDens, suggesting its ability to quantify local heterogeneity degree in neural tissue. These results elucidate the biophysical mechanism underpinning tAD parameters and show the clinical potential of tAD-imaging, considering that both physiologic and pathologic neurodegeneration translate into alterations of WM morphometry and topology.

## Introduction

Living tissues and specifically, brain tissues, are complex biological systems better described by complex systems science (Anderson, 1972; Ngai, 2011), which provides radical new ways of understanding the underlining physics and biology compared to the laws of conventional biophysics. Therefore, new strategies related to diffusion magnetic resonance imaging (DMRI) aspiring to perform early diagnosis and more sensible and specific follow-up of pathologies, should be based on the same aforementioned principles.

In the last two decades, DMRI based on the normal Brownian diffusion, has revolutionized MRI diagnostics and translational MRI studies as it obtains intra-voxel information on tissues by exploiting the diffusion of biological water in the tissues themselves (Jones, 2011). Microstructural information is achieved independently from the image resolution (related to the voxel size) and is limited by the intrinsic resolution l_*D*,_ approximately equal to the square root of the mean square displacement (MSD) of the diffusing protons. MSD = 6DΔ is the mean squared distance traveled by water molecules in a given time interval Δ (or diffusion time), D is the diffusion coefficient, a useful parameter to characterize particle diffusion in the normal case, for which a linear scaling relation between MSD and Δ exists.

In human investigations, DMRI provides a measure of water proton displacement by probing motion at the mesoscopic length scale (l_*D*_ around 10-20 μm), which is orders of magnitude smaller than the macroscopic MRI resolution (typically 1-2 millimeters for clinical MRI scanners). In animal model investigations performed at a very high magnetic field, specific DMRI protocols allow probing tissues with l_*D*_ around the micrometer scale (Shemesh et al., 2013; Porcari et al., 2016; Gao et al., 2020; Müller et al., 2020).

From the point of view of diffusing biological water, neural tissue is a hierarchical multiscale system with different length scales of intracellular and extracellular diffusion compartments, an intricate microvasculature network, submicroscopic traps, and dead space microdomains that transiently entrap diffusing molecules, roughness and barriers, hindering and trapping water diffusion. The normal diffusion of bulk water in tissues is not capable of detecting any of the characteristics above, providing nonlocal, diffusion measurement averaged on the l_*D*_ length scale. As well summarized by Di Tullio et al. (2019) and introduced above, normal or Brownian diffusion is identified by the linear growth in time of the MSD and by the Gaussian shape of the molecular motion propagator. Processes departing from at least one of the above conditions define anomalous diffusion, thus a nonlinear growth in time of the MSD and/or a non-Gaussian displacement distribution.

With its capability to probe tissues with l_*D*_ below the micrometer scale, transient anomalous diffusion (tAD) (Capuani and Palombo, 2020) and its new local parameters would dramatically increase DMRI diagnostic potential in detecting collective, micro and sub-micro architectural changes of human tissues due to pathological damage. Anomalous diffusion (Metzler and Klafter, 2000; Burov et al., 2011; Metzler et al., 2014; Chakraborty and Roichman, 2020) is ubiquitously observed in many complex biological systems, ranging from soft matter, e.g., the cell cytoplasm, membrane (Saxton and Jacobson, 1997; Tolić-Nørrelykke et al., 2004; Golding and Cox, 2006; Zaid et al., 2009; Weigel et al., 2011; Javanainen et al., 2012; Hofling and Franosch, 2013; Honigmann et al., 2013; Jeon et al., 2016; Metzler et al., 2016; Pöschke et al., 2016) and, extracellular space (ECS) (Sykovà and Nicholson, 2008; Sherpa et al., 2014; Nicholson, 2015; Xiao et al., 2015) to the nucleus (Bronstein et al., 2009; Stadler and Weiss, 2017; Pierro et al., 2018) and neuro-physiological systems (Allegrini et al., 2015; Paradisi and Allegrini, 2017).

Different from the normal diffusion, anomalous diffusion is characterized by an MSD of diffusing particles growing nonlinearly in time. MSD ∝Δ^ν^ with ν≠ 1, and ν < 1 for subdiffusion, ν > 1 for superdiffusion (Metzler and Klafter, 2000). There are different well established theoretical frameworks describing anomalous diffusion phenomena, such as the continuous time random walk (CTRW) (Metzler and Klafter, 2000) and the fractional Brownian motion (FBM) (Deng and Barkai, 2009; Magdziarz et al., 2009). These have been corroborated substantially by Monte Carlo simulations together with experimental evidence, mainly obtained with fluorescent spectroscopy (Metzler et al., 2014, 2016; Sherpa et al., 2014; Xiao et al., 2015).

The use of anomalous diffusion parameters quantified using specific DMRI acquisition protocols and its interpretation has often aroused skepticism and misgiving (Jelescu and Budde, 2017; Novikov et al., 2019). Although the derived formulas may accurately fit experimental diffusion weighted (DW) data (Magin et al., 2008; De Santis et al., 2011; Grinberg et al., 2014; Yu et al., 2018), and fractional diffusion equations models (Zhou et al., 2010; Magin et al., 2020; Wang et al., 2021) capture and characterize the multi-exponential features of the signal attenuation, the relation between the postulated dynamical feature and the underlying geometrical structure remains elusive or at most, only suggestive. Further, the fact that the stretched-exponential function, the function type predicted by the anomalous diffusion theory to quantify the superdiffusion and subdiffusion parameters, was used in a qualitative way to fit experimental diffusion-weighted images (DWI) data (Capuani and Palombo, 2020) added confusion to the scenario.

In this paper, we follow the acquisition instructions, the nomenclature, and the anomalous diffusion parameter interpretations of Capuani and Palombo (2020). Specifically: (a) anomalous diffusion in biological tissues is transient, i.e., for very short and very long diffusion times normal diffusion with a finite D_0_ and a finite D∞ (Novikov et al., 2019; Capuani and Palombo, 2020), respectively, is found; (b) transient subdiffusion can be quantified in a multiscale heterogeneous medium characterized by at least three different length scales, and subdiffusion quantifies the local disorder degree (Palombo et al., 2011, 2012, 2013; Capuani et al., 2013); (c) to quantify subdiffusion, DW data from a pulse field gradient (PFG) sequence must be collected varying Δ while keeping gradient strength g constant. Conversely, to quantify superdiffusion, DW data must be collected varying g while keeping Δ constant; (d) no real superdiffusion can be quantified in biological tissues. Using tAD signal representation, pseudo-superdiffusion parameters, which depend on magnetic susceptibility differences Δχ and magnetic inhomogeneities, are quantified (Palombo et al., 2011, 2012; Caporale et al., 2017; Guerreri et al., 2019).

This work aims to elucidate and validate (Jelescu and Budde, 2017) the image contrast and information obtained using the tAD model in white matter (WM) through a direct comparison between different diffusion metrics and histology. Due to the peculiar characteristics of complex tissue that the tAD could highlight, in addition to the conventional parameters describing the geometry of the tissue, such as the axons diameter and the axons density, we quantified some local parameters, such as the effective local density (ELD) that reflects how closely the axons are packed (Comin et al., 2014).

Towards this goal, we compared tAD metrics extracted from subdiffusion (α-imaging) and pseudo-superdiffusion (γ-imaging) in the WM of an excised mouse spinal cord, together with T2 and T2* relaxometry, conventional (normal diffusion-based) diffusion tensor imaging (DTI) (Basser and Jones, 2002) and q-space imaging (QSI) (Callaghan, 1991; King et al., 1997), with morphologic measures resolved by optical microscopy, to determine which structural and topological characteristics of myelinated axons influenced tAD contrast. Three main factors motivated the choice of the sample for this study: (a) in the spinal cord the myelinated fibers, organized in coherent bundles with a certain orientation dispersion, are clearly distinguishable from the gray matter nuclei; (b) the cylindrical geometry of the sample itself leads to a simplification of the diffusion acquisition and description; (c) mouse spinal cord is commonly employed in studies of demyelination.

Structural and topological characteristics (axon diameter, AxDiam, the standard deviation of diameters, SD_*ax.diam*_, axonal density, AxDens and ELD) were extracted from optical images in several WM tracts. Among all the diffusion parameters quantified at 9.4 T, γ-metrics confirmed a strong dependence on magnetic inhomogeneities quantified by R2* = 1/T2* (Palombo et al., 2011, 2012; Caporale et al., 2017; Guerreri et al., 2019) and showed the strongest associations with AxDiam and ELD while α-metrics showed strong associations with SD_*ax.diam*_ and was significantly related with AxDens. QSI-metrics was strongly related to AxDens. DTI-metrics showed non-significant trends with morphology (*P* > 0.05), and none was sensitive to SD_*ax.diam*_. These results elucidate the biophysical mechanism underpinning of tAD parameters and show the clinical potential of tAD-imaging, considering that both physiologic and pathologic neurodegeneration translate into alterations of WM morphometry and topology.

## Materials and Methods

### Sample Preparation

**FIGURE 1:**
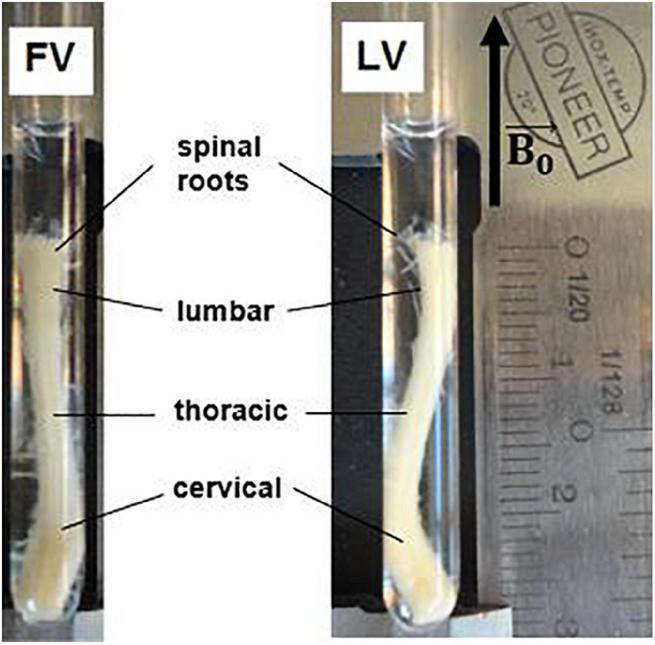
Excised mouse spinal cord in a 5 mm glass capillary for MRI examination. The specimen is about 2.6 cm in length, with maximum cross section of 4 mm × 2.5 mm at the level of the cervical and lumbar enlargements. Different tracts of the spinal cord are indicated. FV = frontal view, LV = lateral view. Black arrow indicates static magnetic field direction.

All applicable international, national, and/or institutional guidelines for the care and use of animals were followed. The mouse spinal cord was extracted after apposite treatment aimed at optimizing fixation, fully described in Ong et al. (2008). A C57 BL6 mouse, (8-9 months, 25-30 mg, Charles River, Wilmington, MA, United States) was anesthetized with an intraperitoneal injection of 10 mg ketamine/1 mg acepromazine per ml, and perfused through the heart with 20 ml of phosphate buffer solution (PBS) and 20 ml of fixing solution with 4% glutaraldehyde and 2% paraformaldehyde in 10 mM PBS. Following fixation, the entire spinal cord was dissected out and post-fixed for at least two weeks in a different fixing solution (2.5% glutaraldehyde and 2% paraformaldehyde in 0.1 M sodium cacodylate). The combination of glutaraldehyde and paraformaldehyde was chosen for its effectiveness in preserving the myelin sheath ultra-structure and intra-axonal cytoskeletal protein (Schwartz et al., 2005). The fixed mouse spinal cord was then inserted in a 5 mm-diameter capillary for the MRI examination (Figure 1).

### Optical Imaging

Optical histologic imaging was performed after MRI at the thoracic and lumbar locations, approximately corresponding to the slices imaged with MRI. The sample preparation followed the protocol described in Sommese et al. (2012). Briefly, the spinal cord was dehydrated in ascending series of ethyl alcohol and then embedded in epon. Semi-thin (1.5 μm) sections of spinal cord were cut with a glass knife, then stained with 1% toluidine blue solution prepared in 1% sodium tetraborate buffer for light microscopy observations.

### Diffusion Magnetic Resonance Imaging Acquisitions

The capillary containing the fixed mouse spinal cord (MSC) was inserted in the 10 mm-diameter bore of a Bruker Avance-400 high resolution spectrometer operating at a magnetic field strength of 9.4 T (maximum gradient strength 1,200 mT/m, rise time 100 μs), so that the ideal cylindrical axis was parallel to the static magnetic field B_0_ (Figure 1). ParaVision 3.0 software was employed for data acquisition. The MSC was scanned along three orthogonal directions, with slice packages placed in the thoracic and lumbar sections. The DWIs were acquired using a Pulsed Gradient Stimulated Echo (PGSTE) sequence, with specific acquisition parameters for γ-imaging, α-imaging DTI and QSI experiments, listed in Table 1. The temperature of the specimen was monitored and kept constant at T = 22°C, with oscillations of +/− 0.5°C, to control for spurious increase of diffusivity due to temperature (Hasegawa et al., 1994).

**TABLE 1:** Acquisition parameters for the Pulse Gradient Stimulated Echo sequences.

	DTI	γ -imaging	α -imaging	q-space imaging
Slice thickness (mm)	1.0	1.0	2.0	0.75
FOV (mm^2^)	4.5 x 4.5			
In-plane spatial resolution (μm^2^)	35 x 35			
Matrix size	128 x 128			
TR (s)	3.5	3.5	(4 - △)	3.5
TE (ms)	12			
Diffusion gradient duration δ (ms)	2			
Diffusion gradient separation **Δ** (ms)	40	40	40, 60, 80, 150, 300, 500, 800	40
Mixing time (ms)	34	34	34, 54, 74, 144, 294, 494, 794	34
Diffusion gradient directions	(1 0 0) (0 1 0) (0 0 1) (1/$\sqrt{2}$ 1/$\sqrt{2}$ 0) (1/$\sqrt{2}$ 0 1/$\sqrt{2}$) (0 1/$\sqrt{2}$ 1/$\sqrt{2}$)	(1 0 0) (0 1 0) (0 0 1)	(1 0 0) (0 1 0) (0 0 1)	(1 0 0)
Effective b-value range for each direction (b_0_, b_*max*_) (s/mm^2^) (effective b-value takes into account the contributions from imaging gradients)	61.2, 1561.2 61.2, 1561.2 61.2, 2142.0 61.2, 1561.2 61.2, 1971.9 61.2, 1971.9	61.2 - 8521.2 61.2 - 8521.2 61.2 - 9496.6	67.9 - 1493.6 67.9 - 1493.6 66.9 - 1491.7	74.7-14171
Effective g value range for each direction (g_0_, g_*max*_) (mT/m)	73.7-372.4 … 73.7-418.5	73.7- 869.9 73.7- 869.9 73.7- 918.4	77.7 - 80.8 77.7 - 80.8 76.9 - 80.7	81.5-1122
Number of b-values (excluding b_0_)	1	16	7	30
Number of averages	8	8	16 (for Δ = 40, 80 ms) 32 (150, 300 ms) 64 (500, 800 ms)	16

#### Diffusion-Weighted Images Data Analysis

##### Diffusion-Weighted Images SNR

The signal to noise ratio (SNR) was computed by considering the ratio between the mean intensity in a selected region of interest (ROI) and the standard deviation of the background intensity. Three different tissues were considered and compared: ‘fluid’ stands for the fixative solution, ‘wm’ stands for the white matter of the lateral column of the spinal cord, and ‘gm’ stands for the gray matter taken in the lateral portion next to the central canal, and not overlapping with it.

##### Diffusion-Weighted Images Denoising

The signal produced from the attenuation due to the random motion of water molecules is affected by noise, especially at high b-values. It is fundamental to provide indications about the SNR of the image, to ensure the reliability of the DWIs data. Among the techniques used to artificially increase the SNR there is data smoothing through 2D spatial filters such as Wiener filter (employing a gaussian kernel). However, this filter changes the stochastic properties of noise, causing data biases, and complicating the fitting procedure of DWIs. Recently, a denoising procedure specific for DWIs was developed and released as an opensource package (Kellner et al., 2016; Veraart et al., 2016). This procedure allows to reduce the noise level, removing the Gibbs artifacts and the RF-spiking, and consequently increases the SNR. The process consists of two steps: first, through Partial Component Analysis (PCA), the noise is reduced with the function ‘dwi-denoise’ (Veraart et al., 2016); in the second step, carried out by the function ‘mrdegibbs’, the Gibbs ringing artifact is removed, as explained fully in Kellner et al. (2016).

### Transient Anomalous Diffusion Metric*s*

#### Pseudo-Superdiffusion: γ-Imaging

The γ metrics (Palombo et al., 2012; Caporale et al., 2017) is extracted from a series of DWI experiments, performed at increasing diffusion gradient strengths *g*, keeping △ constant (Capuani and Palombo, 2020; Table 1.). This technique allows the extraction of γ parameter, quantifying water pseudo-superdiffusion in tissues (Palombo et al., 2012; Caporale et al., 2017). The γ exponent is an adimensional parameter defined in the interval [0, 1], and it is extracted by fitting the stretched exponential function to signal attenuation S(q):


(1) $$S\,\left(q\right)=A\cdot e^{-D_{g\,e\,n}\cdot q^{\gamma }\cdot △}+c$$

with *q*=Υ_*H*_*g*δ (δ, is the gradient-pulse width, Υ_*H*_ is the nuclear gyromagnetic ratio), A and c amplitude and offset, respectively, and D_*gen*_ a generalized diffusion constant. Exploiting the cylindrical approximation, the spinal cord can be imaged along three orthogonal directions (the first one, γ_1_, parallel to the B0-field, the other two, γ_2_ and γ_3_, in the axial plane). Mean-γ (Mγ) is the average of the three exponents; its anisotropy, Aγ, and the longitudinal and radial values (γpar, γort), respectively, parallel and orthogonal to the main axonal fiber orientations, are derived as follows:


(2) $${\text{M}}_{\gamma }=\sum_{i=1}^{3}\gamma_{i}/3$$

(3) Aγ=3⁢[(γ1-Mγ)2+(γ2-Mγ)2+(γ3-Mγ)2]2⁢(γ12+γ22+γ32)

(4) $$\gamma_{p\,a\,r}=\gamma_{1}$$

(5) $$\gamma_{o\,r\,t}=\frac{\gamma_{2}+\gamma_{3}}{2}$$

#### Transient Subdiffusion: α-Imaging

In the α-imaging technique several DWI experiments are performed increasing the diffusion time △ and keeping the diffusion gradient strength g constant (Table 1). The α exponent quantifies water transient subdiffusion in tissues (Capuani et al., 2013; Palombo et al., 2013). It is a dimensionless parameter defined in the interval [0, 1], and it is extracted by fitting the stretched exponential function:


(6) $$S\,\left(△\right)=A\cdot e^{-D_{g\,e\,n}\cdot q^{2}\cdot {△}^{\alpha }}+c$$

to the signal attenuation S(△). By acquiring DWI along three orthogonal directions one may derive the mean α (Mα), the α anisotropy (Aα) and the longitudinal and radial values (α_*par*_, α_*ort*_):


(7) $${\text{M}}_{\alpha }=\sum_{i=1}^{3}\alpha_{i}/3$$

(8) Aα=3⁢[(α1-Mα)2+(α2-Mα)2+(α3-Mα)2]2⁢(α12+α22+α32)

(9) $$\alpha_{p\,a\,r}=\alpha_{1}$$

(10) $$\alpha_{o\,r\,t}=\frac{\alpha_{2}+\alpha_{3}}{2}$$

### Conventional Diffusion and T2* Maps

T2* maps were obtained to investigate the dependence of tAD parameters on magnetic inhomogeneities. T2* weighted images were acquired using gradient echo fast imaging (GEFI) sequences with 15 TE values varying in the range (1.8, 40) ms (TE = 1.8, 2, 4, 6, 8, 10, 12, 14, 16, 18, 20, 25, 30, 35, 40 ms), TR = 1200 ms, matrix 128x128, FOV = 4.5x4.5mm^2^, NEX = 8-16.

To obtain mean diffusivity (MD), fractional anisotropy (FA), radial diffusivity (D_*ort*_) and axial diffusivity (D_*par*_), DWIs along six gradient directions using b = 0, 1000 s/mm^2^ were acquired (Table 1). A mono-exponential model for DW signal decay was used (Basser and Jones, 2002).

#### Investigation of the Dependence of γ-Metrics From Magnetic Field in-Homogeneities

With the double purpose of verifying the dependence of γ-metrics on magnetic field in-homogeneities, and of verifying that γ-metrics for the fluid in the capillary approached unity (as predicted by theory) we considered five ROIs, other than those selected on the optical microscopies. By thresholding the MD map of axial and sagittal slices of the mouse spinal cord, five ROIs were extracted, comprising: the medium surrounding the spinal cord, the gray-matter, the interface between white matter, and white matter more or less densely packed. Mean values of γ-metrics and R2* (the inverse of T2*) were extracted, and the linear regression was evaluated.

#### Q-Space Imaging (QSI)

Unlike diffusion tensor imaging, QSI identifies the molecular diffusion probability density function without the need to assume a normal diffusion with Gaussian distribution processes.

In QSI, the attenuation of the echo signal is recorded as a function of the q-value, defined as $\left(\frac{1}{2}\,\pi \right)\,\Upsilon_{H}\,g\,\delta$ (Callaghan, 1991). The echo attenuation S(q) along an arbitrary gradient direction is the Fourier Transform of molecular displacement probability function, P(r,Δ):


(11) $$S\,\left(q\right)=\int P\,\left(r,△\right)\,e^{i\,2\,\pi \,q\cdot r}\,dr$$

When a single gradient direction is used, the one-dimensional displacement probability function may be referred to as the displacement profile. The 1D-QSI displacement profile has a particularly simple interpretation if the gradients are applied perpendicularly to the axon fibers, in presence of a tubular geometry as in the case of spinal cord. The displacement profile can be fitted with a Lorentzian curve, centered in 0:


(12) L⁢(x,y0)=1π⋅y0x2+y02+c

With c the offset term, and y0 equal to half the full width at half maximum (FWHM). The FWHM correlates with the scale of restrictions which, in WM, is the mean axon diameter averaged over the imaging volume (Ong et al., 2008; Ong and Wehrli, 2010; Cohen et al., 2016).

If the range of *q*-values is limited, the ‘low q-value approximation’ (Ong et al., 2008; Ong and Wehrli, 2010) can be used. It consists in a bi-exponential model where Zics and Zecs represent the width of the intra (ICS) and extra (ECS) cellular space, respectively:


(13) $$S\,\left(q\right)=f\cdot e^{-2\,\pi^{2}\cdot q^{2}\cdot Z_{e\,c\,s}^{2}}+\left(1-f\right)\cdot e^{-2\,\pi^{2}\cdot q^{2}\cdot Z_{i\,c\,s}^{2}}$$

with f the fraction of ECS, and 1-f the fraction of ICS, including the myelin compartment.

### Histological Investigation and Quantitative Analysis

**FIGURE 2:**
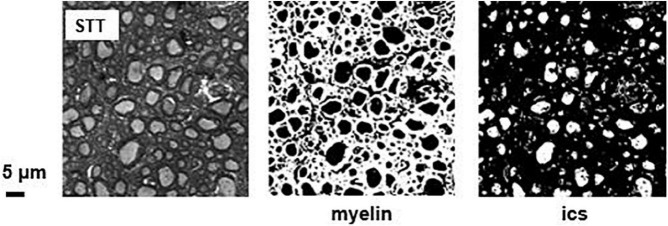
Representative optical image segmentation. Optical image of the spino-thalamic tract (STT) in the thoracic segment at the level T3/T4, with the result of the threshold-based segmentation algorithm, yielding binary masks for myelin and intracellular space.

A specific software for the extraction of morphologic and topologic parameters from 2D optical microscopy images of MSC was developed in MATLAB R2016b (MathWorks, Inc., Natick, MA, United States) (see Supplementary Appendix 1). Briefly, the script takes in input selected 2D optical microscopies, performs pre-processing (Wiener filter of width 0.2 μm), segmentation, object recognition, applies selection rules, and provides the desired quantitative measures in output. Optical microscopies were digitized and segmented (Figure 2) into four tissue classes, based on thresholds intensity: intracellular space (ICS), extracellular fluid (ecs), myelin (myel), and the pixels belonging to other structures/cells not ascribable to any of the other three types (other). Indicating with f_*xxx*_ the fraction of area occupied by the specific tissue class, the following normalization rule was considered:


(14) $$f_{i\,c\,s}+f_{e\,c\,s}+f_{m\,y\,e\,l}+f_{o\,t\,h\,e\,r}=1$$

The squared pixel dimension of each image is 0.033 μm × 0.033 μm. A pixel-wise adaptive Wiener method based on statistics estimated from a local neighborhood of each pixel was applied prior to the segmentation. The mask associated to the ics component was polished through surface, circularity and uniformity thresholds, as described in literature (Comin et al., 2014): briefly, the algorithm discarded: those objects with an area smaller than a specific value defined by the user, presumably representing speckles and non-axons spots; the objects for which the ratio of the perimeter to the square root of the area was larger than 6.2, corresponding to elongated ellipses with b > 6a, with a and b the semi-minor and semi-major axes of the ellipse, respectively; the objects with a ratio σ/μ > 0.5 of the standard deviation σ and the mean intensity μ, assumed to contain debris or multiple tissue types.

**FIGURE 3:**
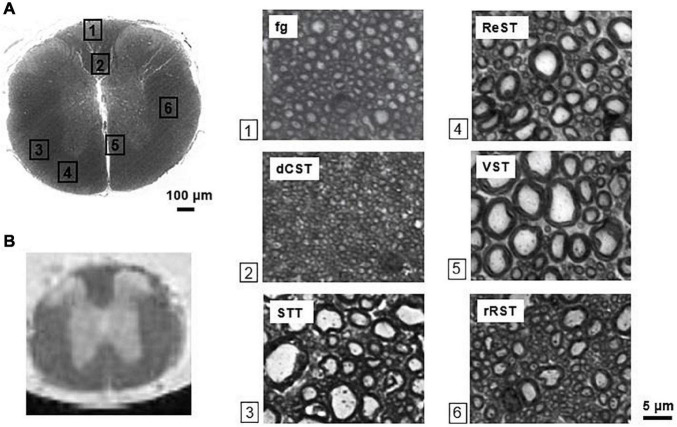
Mouse spinal cord histology. **(A)** Optical image of the thoracic segment at the level T3/T4, with magnifications (×100) of selected white matter tracts. The scalebar is indicated (bar = 5μm for each magnification). **(B)** T2-weighted image showing the corresponding slice. The ventral side of the cord is adjacent to the capillary walls. Fg = funiculus gracilis, dCST = dorsal cortico-spinal tract, STT = spino-thalamic tract, ReST = reticulo-spinal tract, VST = vestibulo-spinal tract, rRST = right rubro-spinal tract.

Six ROIs were manually drawn encompassing six WM tracts: funiculus gracilis (fg), dorsal-Corticospinal tract (dCST), rubrospinal tract (RST), spino-thalamic tract (STT), vestibulo-spinal tract (VST), reticulo-spinal tract (ReST) (Figure 3). In each region, axon diameter (Ax. diam.), diameter SD (SD ax. diam), axon density (Ax. Dens.) and effective local density (ELD) as extracted in Comin et al. (2014) were estimated. The Ax Dens was derived from the ratio between the fraction of area occupied by axolemmas, and the area of an axon with average diameter computed over the ROI, and approximated to a circle. Effective local density (Comin et al., 2014) is a modified version of the axon density, and reflects how closely the axons are packed. It is an estimate of the density of axons around the axons, ignoring axon-free regions. A high value of ELD indicates that axon free-areas are clustered, while a low ELD indicates that axon-free areas are spread out. Different from the standard axonal density, which does not consider the various obstacles in the ECS not recognized as axons and not passing the selection rules, the ELD accounts for the area not occupied by axons.

Average values across the ROIs and the specimens where considered, with their standard deviations, using the masks provided by the segmentation procedure. The analysis was carried out by two different operators. The average histological characteristics of the considered white matter tracts are listed in Table 2.

**TABLE 2:** Average regional histologic characteristics of WM tracts.

	dCST	fg	rRST	ReST	STT	VST
Axon Diameter (μm)	0.82 ± 0.04	0.93 ± 0.06	1.23 ± 0.07	1.50 ± 0.08	1.53 ± 0.05	2.2 ± 0.19
Diameter SD (μm)	0.49 ± 0.09	0.48 ± 0.05	0.75 ± 0.11	0.82 ± 0.12	0.79 ± 0.20	0.81 ± 0.13
Axon Density (10^5^/mm^2^)	3.63 ± 0.49	3.04 ± 0.21	1.91 ± 0.36	1.56 ± 0.26	1.48 ± 0.11	0.69 ± 0.22
Effective local density (x10^–3^)	3.8 ± 1.8	3.4 ± 1.3	2.7 ± 1.3	2.2 ± 0.3	3.0 ± 1.8	1.6 ± 1.1

### Statistical Analysis

Pearson’s correlation coefficients were computed between the diffusion metrics and the histological characteristics. *P* < 0.05 was used to demonstrate statistical significance.

### Extraction of d-μMRI Parametric Maps

Conventional DTI analysis was performed by means of FSL 5.0 DTIFIT routine (FMRIB Software Library v5.0, FMRIB, Oxford, United Kingdom), which returns the maps of Mean Diffusivity (MD), Fractional Anisotropy (FA), and the 3 eigenvalues (λ_1_, λ_2_, λ_3_) and eigenvectors (V_1_, V_2_, V_3_) from the diagonalization of the tensor.

The PGSTE data were fitted to the theoretical models of γ-imaging, α-imaging and q-space imaging (respectively, represented by Eq. 1, Eq. 6, Eq. 12, and Eq. 13) using custom MATLAB scripts (MATLAB R2016a), employing a non-linear least square estimation procedure with ‘trust-region reflective’ algorithm for minimization, and parallel computing.

The non-linear fit expression used for γ-imaging was:


(15) $$\frac{S\,\left(q\right)}{S_{0}}=p_{1}\cdot e^{\left(-p_{2}\cdot q^{p_{3}}-D_{0}\,q_{0}^{2}\right)\cdot △}+p_{4}$$

As suggested by Jones and Basser’s paper (Jones and Basser, 2004), we included in the fitting procedure a separate “noise” parameter, the p_4_ parameter, to take the noise floor into account. We fixed the offset p_4_ = 0.15, because the parameterized additive constant p_4_ converged to 0.15 for different initializations of p_4_ in the non-linear fit with four parameters performed in previous fit tests. The other three-parameters returned the multiplicative constant p_1_, the generalized diffusion constant (p_2_ = D_*gen*_) and the value proportional to the gamma-exponent (p_3_ = 2γ) for each of the three orthogonal directions on a voxel-by-voxel basis, taking about 6 minutes for each slice on a 2.6 GHz 4-cores machine with 16.0 GB RAM. The signal attenuations were thus normalized to the background signal, and to the image without diffusion weighting (S_0_). The parameters were initialized, respectively, to [1, 1*10^–10^ 1.8], with lower bounds [0, 0, 0] and upper bounds [Inf, Inf 2].

For the α-imaging there is the necessity to normalize the signal attenuations to the background and to the receiver gain (RG), which increases with noise, thus with the observation time (RG = 64, 64, 64, 64, 421.147, 855.654, 2801.08); furthermore, the use of a logarithmic fit provides inferior norm of residuals. The non-linear fit expression used for α-imaging was thus:


(16) ln⁢(Sd⁢(△,q))∼ln⁢(p1)-p22⁢q02⋅△p3

where ‘*d*’ denotes the direction of acquisition (*d* = 1,2,3), p_1_ is a multiplicative constant, p_2_ represents the generalized diffusion constant in the sub-diffusive regime, p_3_ represents the α exponent we are looking for, $q_{0}^{2}$ is the mean q-value, considering all the used mixing times (which is not expected to vary among experiments, since we selected carefully the intensity of diffusion gradient to be constant, with δ fixed). The output provided by the scanner is the b-value, thus we derived q-value from: $q=\sqrt{\frac{b}{4\,\pi^{2}\,\left(△-\frac{\delta }{3}\right)}}$. The parameters were initialized as follows: (p1_0_, p2_0_, p3_0_) = [max(S_*d*_), MD, 0.9], with lower bounds [0, 0, 0.5] and upper bounds [Inf, Inf 1.1]. For the α-imaging it was necessary to segment the spinal cord, fitting the WM and the gray matter (GM) + fluid separately. The generalized diffusion constant was initialized to the value of MD, in the order of 10^–3^/10^–4^ mm^2^/s. The required time for the fitting procedure was about 2.5 minutes for each slice on a 2.6 GHz 2-cores machine with 16.0 GB RAM.

Because PGSTE was used, to avoid bias in α-metric estimation, the relaxation time T_1_ of the fixed mouse spinal cord at 9.4 T was previously measured. It was about 600 ms in the GM and 760 ms in the WM. Therefore, in a preliminary step, to take into account the T_1_ signal decay during the mixing time TM∼Δ, we use both Eq. 15 and a modified Eq. 15 setting *p*_1_⋅*e*^−Δ/*T*1^ instead of p_1_ to perform the fitting procedure. We found that both fitting procedures provided the same α value, and slightly different p_1_ and p_2_ parameters.

For the q-space imaging the signal attenuations were normalized to the background and to S0. The non-linear fit expression used in the case of ‘low q-value approximation’ was:


(17) S⁢(q)S0=p1⋅e-2⁢π2⁢p22⁢q2+(1-p1)⁢e-2⁢π2⁢p32⁢q2+p4

To take into account the noise floor (Jones and Basser, 2004), we fixed the offset p_4_ = 0.2, because the parameterized additive constant p_4_ converged to 0.20 for different initializations of p_4_ in previous tests of non-linear fit with four parameters. The other three-parameters returned the relative fraction of the extracellular compartment (p_1_ = proportional to f_*ecs*_), the mean square displacement in the extracellular compartment (p_2_ = Z_*ecs*_) and of the intracellular compartment p_3_ = Z_*ics*_. The parameters were initialized, respectively, to [max(S (q)*0.7), 5*10^–6^, 0.15], with lower bounds [0, 0, 0] and upper bounds [2, 10^–2^,10^–2^]. The fitting procedure requires about 3 min for each slice on a 2.6 GHz 2-cores machine with 16.0 GB RAM. As in the α-imaging, it is better to segment the tissues, and let the fit work on WM and GM separately. The multiplicative constant in p_1_ represents the f_*ecs*_, and it is ascribable to the fractions of ECS. The initializations to 0.3 and 0.7 provide the same norm of residuals, causing an indetermination in the right estimate of f_*ecs*_.

By mirroring the signal attenuations with respect to 0, and performing a Fourier Transform (using “dft.m” function in MATLAB, and a customized function called “dftgh6.m”), it was possible to derive the Lorentzian shaped pdf of displacements. This curve, which was truncated at 11 values, because of fluctuating tails due to noise (we are in the short pulse gradient approximation, SPGA, yet we have too little q_*max*_ to achieve the acceptable resolution), was fitted to the Lorentzian described in Eq. 12, that is:


(18) L⁢(x)=p1+1π⋅p2x2+p22

Where p_1_ represents an offset, p_2_ is the parameter controlling the width of the curve (the FWHM of which is 2p_2_). The parameters were initialized as [1, 1], and set free to vary within lower bounds [−Inf 0] and upper bounds [Inf, 10^–4^]. The fitting procedure requires about 2.5 minutes for each slice on a 2.6 GHz 2-cores machine with 16.0 GB RAM.

## Results

### Diffusion-Weighted Images Acquisitions: SNR and the Effect of Diffusion-Weighted Images Denoising

The reliability of tAD maps depends on the SNR of DWIs at each b-value. We found that SNR of acquired DWIs was greater than 10 in both GM and WM and in each diffusion gradient direction and strength used.

**FIGURE 4:**
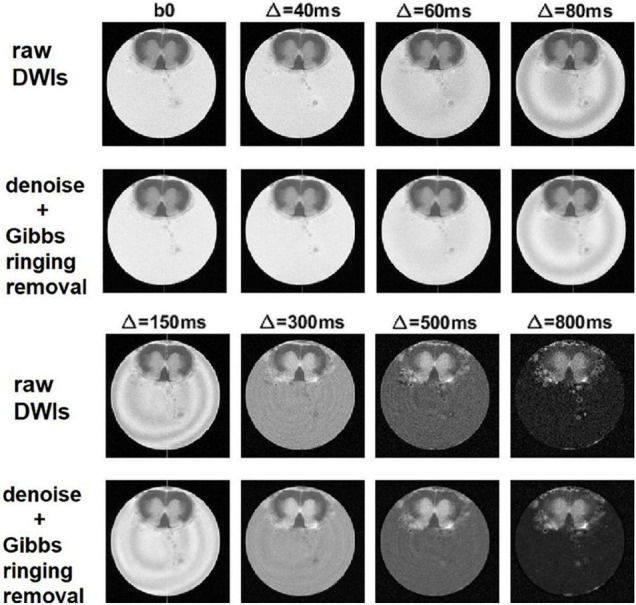
Denoising and removal of Gibbs ringing artifact in DWI used to obtain α-imaging. Comparison between the raw DWIs and the DWIs after denoising and the removal of Gibbs ringing artifact, for the lumbar slice of the spinal cord, considering the DWIs acquired along z-direction and using increasing diffusion time, **Δ**.

**FIGURE 5:**
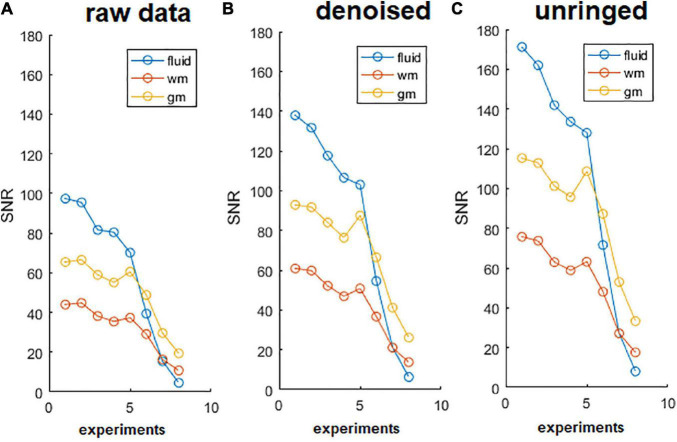
Signal to noise ratio (SNR) of diffusion-weighted images (DWIs). SNR of DWIs collected for α-imaging plotted against the b-values (b0 plus other 7 b-values, in the range 67.9, 1493.6 s/mm^2^) for DWIs acquired with the diffusion gradient acting along the z (longitudinal) direction with △ ranging from 40 ms to 800 ms. **(A)** Raw data, before the noise removal; **(B)** data after the use of dwidenoise function; **(C)** data after the removal of Gibbs ringing artifact. SNR computed in the unringed DWIs is the highest for each of the three tissues (fluid = capillary solution; wm = white matter, right column; gm = gray matter, right ventral horn), up to the longest diffusion time. This comparison was made considering the lumbar section of the spinal cord.

The effect of denoising on raw DWIs was examined. At a visual inspection, the denoised images are less grainy compared to the raw data and the RF-spiking line has disappeared. What is left untouched, is the spiral-like artifact consisting in white/gray stripes, that is not attributable to Gibbs ringing artifact, and thus not corrected by the type of processing used here. However, this artifact affects only free water in the capillary (Figure 4). The denoised images show higher SNR (Figure 5), which is double the SNR computed in raw data.

### Parametric Maps

**FIGURE 6:**
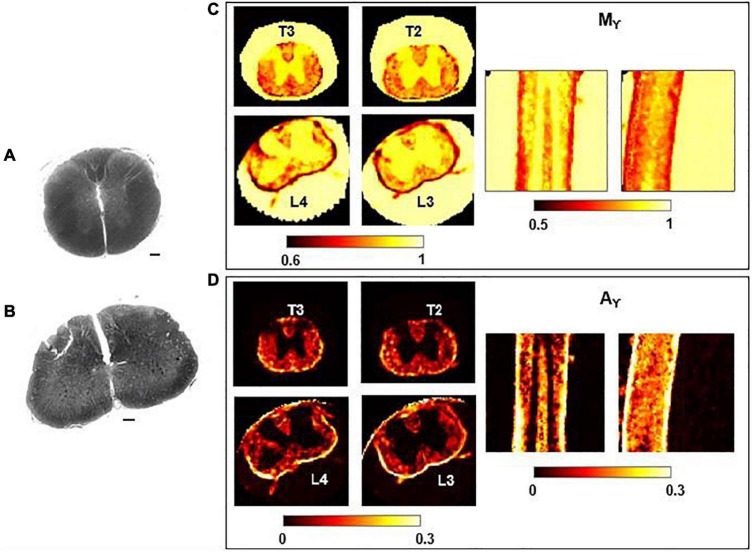
Histology and γ-imaging parametric maps. Optical images of the thoracic segment at the level T2/T3 **(A)** and of the lumbar segment at level L3/L4 **(B)**. Scalebar is 100 μm. **(C,D)** Parametric maps of mean-γ (Mγ) and γ-anisotropy (Aγ) for the corresponding thoracic and lumbar segments. Axial, coronal and sagittal views are shown, from left to right.

**FIGURE 7:**
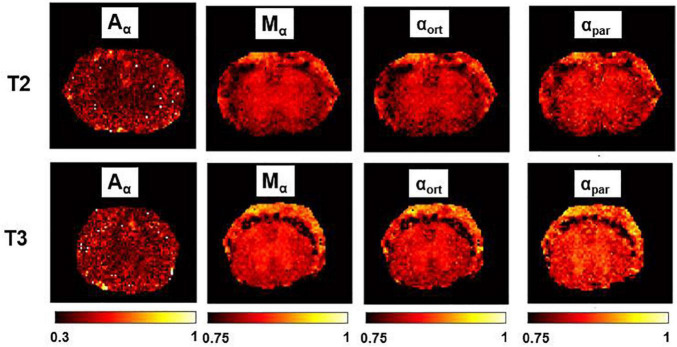
α-imaging parametric maps. Transient subdiffusion maps of thoracic segment at the level T2/T3. Parametric maps of α-anisotropy (Aα), mean-α (Mα), α orthogonal to the WM fibers (α_ort_), and α parallel to the WM fibers (α_par_). α-anisotropy is shown with a different colorbar. The parametric maps are extracted from the axial slices in the thoracic segments T2 (top row) and T3 (bottom).

**FIGURE 8:**
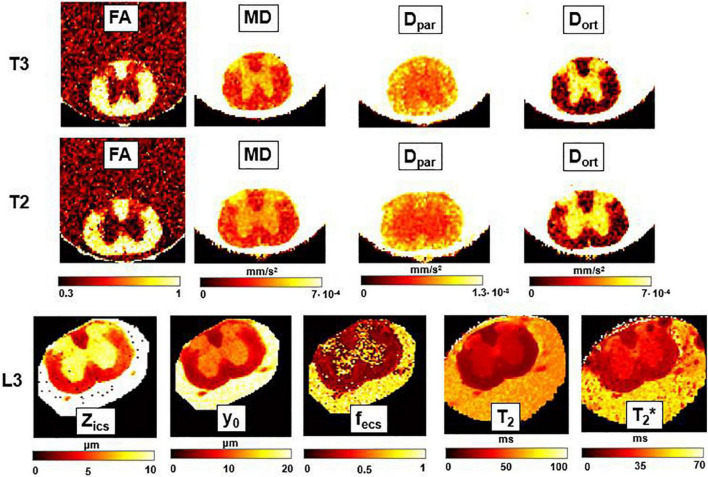
DTI, T2-relaxometry and Q-space imaging. Parametric maps obtained from DTI (fractional anisotropy, FA; mean diffusivity, MD; longitudinal diffusivity, Dpar; radial diffusivity, Dort), Q-space imaging (Zics, representing the width of the intra-cellular space, y0, the FWHM of the Lorentzian, and f_*ecs*_, fraction of extracellular space) and T2 and T2* maps, for three axial slices corresponding, respectively, to the thoracic sections T3 and T2, and to the lumbar section L3. FA and f_*ecs*_ are unitless, while for other parameters the unit of measurement is indicated near the colorbar.

In Figure 6, Mγ, Aγ maps and optical images of the same thoracic and lumbar segments of mouse spinal cord are displayed. Please note that the pseudo-superdiffusion maps (γ-maps) highlight boundaries and barriers between tissues with different magnetic susceptibilities. As expected, the results reported here, confirm the central role of the local Δχ in providing γ contrast, as already observed and described (Palombo et al., 2012; Caporale et al., 2017). In Figure 7, an example of transient subdiffusion in thoracic segments of mouse spinal cord is reported, whereas in Figure 8 conventional DTI maps together with QSI maps and T2 and T2* maps are displayed. It is possible to notice that WM and GM are weakly contrasted in the α compared to the γ, QSI and DTI parametric maps, showing a different image contrast.

### tAD-Metrics Relation With Magnetic Inhomogeneity

**FIGURE 9:**
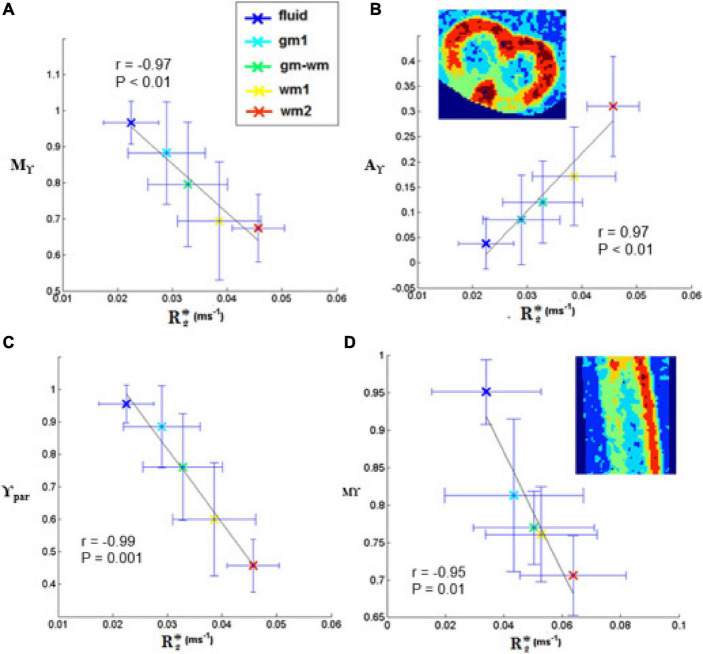
Plots of γ-imaging metrics as a function of R2*. Mean-γ (Mγ), γ-anisotropy (Aγ) and longitudinal γ (γ_*par*_) plotted as a function of R2* in axial **(A-C)** and in sagittal slices **(D)**. The tissue was divided in different regions by applying thresholds on the mean-diffusivity map. The distinct regions are listed in the legend and comprise: the medium surrounding the spinal cord (fluid), two ROIs in the white matter (wm1, wm2), one in the gray matter (gm1), and one at the interface (gm-wm). The Pearson’s correlation coefficient is indicated in the box, together with the level of significance, P. The black line represents the linear fit.

No significant linear correlation was found between R2* mean values obtained in each of the five selected regions (from fluid to GM and WM) and transient subdiffusion parameters (α-metrics). Conversely, a significant linear correlation was found between R2* and pseudo-superdiffusion parameters (γ-metrics), as showed in Figure 9, in complete agreement with previous investigation performed in materials (Palombo et al., 2011, 2012) and human brain tissues (Caporale et al., 2017; Guerreri et al., 2019).

### Linear Correlations Between DTI, QSI, tAD Diffusion Metrics and Histology

The Pearson’s correlation coefficients of the linear correlations between the diffusion-MRI metrics and the histologic features are listed in Table 3.

**TABLE 3:** Linear correlations between DTI, QSI, tAD diffusion metrics and histology.

	Axon Diameter	Diameter SD	Axon Density	Effective local density
MD	−	−	–0.79	−
FA	–0.68	−	0.56	0.78
D_*ort*_	0.80	−	–0.77	–0.81
D_*par*_	−	−	−	−
Mγ	0.98****	0.88*	−0.83*	−0.92**
Aγ	−0.95***	−0.89*	0.80^∧^	0.90*
γ _*ort*_	0.86*	0.84*	–0.78	−0.94***
γ _*par*_	0.95***	0.77	–0.75	−
Mα	−0.82*	–0.76	0.68	−
Aα	–0.72	–0.64	0.83*	−
α_*ort*_	−	−	−	−
α_*par*_	−0.80^∧^	−0.90*	0.85*	−
Z_*ics*_	0.77	0.88*	−0.89*	−0.87*
Z_*ecs*_	−0.83*	−	−	−
FWHM	0.72	0.85*	−0.84*	−0.84*

*P < 0.05, **P < 0.01, ***P < 0.005, ****P < 0.001, ^∧^P = 0.055; ort = orthogonal; par = parallel; ics = intra-cellular space; ecs = extra-cellular space.

None of the DTI parameters showed significant correlations with histological parameters. Dort showed a positive, and FA a negative trend with the axonal diameter. Compared to other parameters, however, Mγ, and γ_*par*_ showed the strongest positive correlations (with *P* < 0.001 for Mγ) with the axonal size (AxDiam). Conversely, Mα and α_*par*_ were inversely related (with significant P for Mα) to the axonal size.

None of the DTI-metrics showed significant correlations with the SD of axons distribution, whereas Mγ, Aγ, γ_*ort*_, α_*par*_, Zics and FWHM were related with the dispersion of axonal size.

Among the DTI parameters, Dort showed a negative trend with the derived axonal density, matching with the results by Schwartz et al. (2005), relatively to the transverse (radial) apparent diffusion coefficient and axons count. α and γ-metrics were also related to axonal density, with correlations approaching significance, and *P* < 0.05 for Mγ, Aα and α_*par*_. Based on the results in Table 3, the parameters which best reflected the derived axonal density are the ones obtained with q-space imaging (Zics and FWHM).

We also considered the correlations with the ELD. While Dort showed a negative trend with ELD (*r* = −0.810, *P* > 0.05), γ-metrics (in particular Mγ and γort) and QSI metrics (FWHM and Z_*ics*_) showed strong negative correlations (*r* = −0.92, *P* < 0.01 for Mγ; *r* = −0.94, *P* < 0.005 for γort; *r* = −0.84, *P* < 0.05 for FWHM; *r* = −0.87, *P* < 0.05 for Zics). No correlation was found between α-metrics and ELD.

## Discussion

The development of new advanced DMRI models to improve the diagnostic potential of diffusion MRI is intrinsically limited, since DMRI is an indirect measure of medium microstructure and relies on inferences from models and estimation of relevant diffusion parameters (Jelescu and Budde, 2017). The use of anomalous diffusion parameters to increase the sensibility and specificity of DMRI investigations has often aroused skepticism and misgiving (Jelescu and Budde, 2017; Magin et al., 2020; Wang et al., 2021). Although the derived formulas may accurately fit experimental DW data, the relation between the postulated dynamical features and the underlying geometrical structure remains elusive or at most only suggestive. Therefore, efforts to validate parameters estimated by anomalous diffusion models with complementary techniques such as optical microscopy of fixed tissue specimens are high desirable (Jelescu and Budde, 2017).

In this work, in an attempt to explain the underpinning biophysical mechanisms and validate parameters of tAD, we analyzed excised tissue of mouse spinal cord using different diffusion protocols already known and validated two different protocols to quantify pure subdiffusion parameters (α-metrics) and pseudo-superdiffusion (γ-metrics). The extracted parameters were compared and correlated to histological parameters extracted by optical microscopy.

The discussion is organized as follows. First, we checked the validity of the parameters AxDiam, SD_*ax.diam*_, AxDens, and ELD extracted from the histological images that we carried out by developing a dedicated software (the details are reported in Supplementary Appendix 1). Subsequently, we verified the strong dependence of the γ-metric on the magnetic field in-homogeneities that we had already observed leading us to define the γ-metric not as a real anomalous diffusion metric, but as a metric that exploits the signal representation of the superdiffusion signal decay to provide useful parameters for the discrimination of different tissue features. We then discussed the results related to the correlations between diffusion metrics and histological parameters, providing an interpretation of the observed phenomena. Finally, we focused on the correlations observed with a new parameter, ELD, which should better express the characteristics of brain tissues described as a complex system, better than conventional structural parameters.

### Histological Parameters

To quantify AxDiam, SD_*ax.diam*_, AxDens, and ELD we previously quantified the ECS, ICS and the myelin fraction. All our morphological parameters were compared with those derived by Ong et al. (2008) and Ong and Wehrli (2010) extracted from the same type of mouse spinal cord, using Transmission Electron Microscopy (TEM) images, with a resolution of 0.1 μm × 0.1 μm in the histology, and a pixel resolution of 0.27 μm × 0.27 μm in the segmented images. Comparing ECS, ICS and myelin fractions through a *z*-test, we concluded that the measured fractions of occupied area are in agreement with literature, except for the fractions of ICS and ECS in dCST. However, the morphological parameters derived by Ong are referred to the cervical section while we have investigated thoracic-lumbar tract. Our morphological derived parameters AxDiam, SD_*ax.diam*_, AxDens showed significant positive correlations with those derived by Ong et al. (2008) and Ong and Wehrli (2010) (see also Supplementary Figure 5).

Regarding the estimation of the ELD parameter, we found only one paper in literature, in which ELD is estimated in a monkey brain. Our results show that ELD values are two orders of magnitude smaller than those calculated in the paper by Comin et al. (2014). In our opinion, the difference is due to the different characteristics of the monkey brain tissue compared to that of the mouse spinal cord and to the different experimental conditions.

### Dependence of the γ-Metric on the Magnetic Field in-Homogeneities and on Axonal Size

In this paper, we found again a strong linear correlation between γ-metrics and magnetic susceptibilities quantified by R2*. This result confirms the observations reported in previous papers obtained in packed polystyrene beads (Palombo et al., 2011, 2012), and in *in vivo* human brains (Caporale et al., 2017; Guerreri et al., 2019). In the presence of a static magnetic field, B_0_, differences in magnetic susceptibility (Δχ) between adjacent diffusion compartments generate a different amount of magnetization due to the relation: M = χB_0_. Therefore, local magnetic inhomogeneity generated by ΔχB_0_ are found at the interface between different tissues. In the mouse spinal cord, a local Δχ between ECS and axon myelin is a source of image contrast in WM region (Figure 6) that appears more heterogeneous compared to MD map (Figure 8). Importantly, all values of γ-metrics parameters are inversely correlated with magnetic susceptibilities (Figure 9) and directly correlated with AxDiam (Table 3). In particular, Mγ assumed higher values in parallel to higher axon diameters and lower values when quantified in WM characterized by smaller axon diameters (see Supplementary Appendix 2). The fact that Mγ is positively related to the axonal size may be linked to microtubules and neurofilaments being progressively more dispersed inside the axolemma, which reduces the Δχ experimented by spins during water displacements.

### Correlations Between tAD Diffusion Metrics and Histological Parameters

Mα is inversely related to the axonal size (Table 3), probably because the ROIs with the largest axons (VST, for example), are those with the highest heterogeneity in the axon size distribution. Indeed, in previous works performed on monodispersed and polydisperse polystyrene microbeads in water (Palombo et al., 2011, 2013), Mα showed a dependence on bead size distribution, assuming higher values in more homogeneous bead size samples and lower values in more heterogeneous bead sizes. To corroborate this interpretation, we found that α_*par*_ is inversely correlated with SD_*ax.diam*_. This feature can highlight the ability of α measured along the axon direction, to highlight packing correlation length for neuronal fibers, as well as the degree of structural disorder along the neurites already supposed in time dependence diffusion, D(t), studies (Novikov et al., 2019). Moreover, our result is in agreement with observations reported in a recent study on mice corpus callosum (Gatto et al., 2019) where a decrease in α values was associated with an increase of longitudinal axons tortuosity. γ-metrics, Mγ is inversely related to AxDens (Table 3). In fact, as axonal density increases, the local susceptibility differences between myelin and ECS decrease, and therefore the Mγ value increases, in accordance with the results reported in Figure 9 and in the previous section.

The AxDens is derived from the ratio between the fraction of area occupied by axolemmas, and the area of an axon with average diameter computed over the ROI, and approximated to a circle. Based on the results listed in Table 3, the parameters which best reflect the axonal density are those obtained with q-space imaging model (Z_*ics*_ and FWHM). However, α_*par*_ showed a positive correlation with AxDens in agreement with the aforementioned elucidations related to α-metrics features. As axonal density increases, local heterogeneity decreases and therefore the value of α increases, as suggested in Palombo et al. (2013).

### Effective Local Density

With the idea of quantifying histological parameters useful for better describing tissues as complex systems, we have quantified the ELD parameter. Differently from the standard axonal density, which does not consider the various obstacles in the ECS not recognized as axons, ELD accounts for the area not occupied by axons. In a study involving rhesus monkeys, ELD was able to discriminate between youth and elderly based on the axonal density of white matter in the fornix (Comin et al., 2014).Unexpectedly, we did not observe any correlation between the α-metric parameters and ELD. Conversely, γ-metrics (in particular Mγ and γort) and QSI metrics (FWHM and Zics) showed strong negative correlations with ELD (Table 3). We conjecture that local magnetic field in-homogeneities may be responsible for these results.

### Limits to Take Into Account

This study has some limitations. First, we assumed that the anomalous diffusion reference frame coincided with the DTI reference frame, and we considered a set of three orthogonal directions (one parallel to the magnetic field and the main orientation of the myelinated fibers in the spinal cord, the other two in the orthogonal plane) to characterize the mean value and the anisotropy of the γ and α parameters. This first order approximation was used in our previous works (De Santis et al., 2011; Caporale et al., 2017; Guerreri et al., 2019), and the resulting scalar invariant metrics provided complementary information with respect to DTI-metrics. However, provided that the DW acquisition is performed in a sufficient number of directions (at least 12, 3 for the stretched exponent, 3 for the effective anomalous diffusion coefficient, and 6 to define the anomalous diffusion reference frame), it would be possible to overcome the first order approximation and to derive the intrinsic anomalous diffusion reference frame. Obviously, this would involve having a greater number of parameters to estimate and therefore acquiring much more experimental data.

A second limitation of this study is that we ignored the orientation distribution of myelinated fibers. Studies on excised and *in vivo* human spinal cord showed a certain orientation dispersion of the neurites. In the paper by Grussu et al. (2016), healthy human spinal cord specimens at the upper lumbar and thoracic level are examined using optical imaging and directional statistics to map the orientation dispersion of the neurites. The orientation dispersion (OD) quantifies how parallel the neurites are to each other. In another work, Grussu et al. (2015) used multi-shell diffusion MRI with the NODDI model to estimate the OD index (ODI) in a human cervical spinal cord *in vivo*. Interestingly, OD reduction can be found in multiple sclerosis lesions with loss of myelin, indicating a less complex neurite architecture (Grussu et al., 2017). The orientation dispersion of neurites can be modeled by a distribution function, taken from directional statistical models, such as Watson, Von Mises, or Gaussian, and their weighted versions (Grussu et al., 2016). However, in our work we assumed that the orientations of the WM fibers were non-dispersed, that is, the fibers were parallel to each other. This can potentially bias the estimated anomalous diffusion metrics, and even reduce the sensitivity of the parameters towards the underlying microstructure. On the other hand, we note that the same assumptions are implied in the derivation of the conventional DTI metrics. Further studies on the impact of the OD on the quantification of γ and α parameters are warranted. In this regard, a comparison between the ODI index and γ-metrics in the human brain was recently highlighted (Guerreri et al., 2019), whereas a first investigation of γ-metrics dependence on neurite orientation distribution can be found in paper (Caporale et al., 2017).

Moreover, we did not take into account the presence of crossing fibers. In Lundell et al. (2011) work, excised cervical spinal cords of vervet monkeys are examined with high angular resolution diffusion imaging (HARDI). Collateral fibers are found in the dorsal and ventral horns, and in the lateral WM, mainly towards the cervical enlargement. Another method to resolve the complex microarchitecture of neurites in the rat spinal cord (and brain) (Özarslan et al., 2006b) highlighted the presence of crossing fibers, where the ventral roots penetrate the WM. Therefore, in our study, we cannot exclude that crossing fibers at the level of the ventral spinal roots have contributed to and partially contaminated the signal in the voxels belonging to certain WM tracts, such as the reticulo-spinal tract and the spino-thalamic tract.

Other papers by Özarslan et al. (2006a,2012) showed results related to sub-diffusion maps in the rat hippocampus obtained using different gradient directions to account for tissue anisotropy. However, the concomitant use of different q values (i.e., g strength change) together with the variation of the diffusion time may have compromised the sub-diffusion contrast.

## Conclusion

In conclusion, in this work, we quantified tAD parameters representing pure subdiffusion (α-metrics) and pseudo-superdiffusion (γ-metrics) in an excised mouse spinal cord to elucidate how tissue features affect tAD quantitatively. Toward this goal, accurate histology of the mouse spinal cord was performed after DMRI scanning to directly compare tAD parameters with geometrical and topologic parameters extracted by optical microscopy. The results discussed here confirm the strong dependence of the γ-metrics on magnetic in-homogeneities already suggested in previous papers performed on heterogeneous samples and on the human brain (Capuani and Palombo, 2020). On the other hand, α-metrics results corroborated by histological validation suggest the potential of pure subdiffusion parameters to highlight new information, complementary to conventional DMRI investigation to characterize complex biological systems. This work suggests that α-metrics may quantify the local heterogeneity degree in neural tissue.

Although with the limitation of having used a simplified model of parallel, and not dispersed axons in the spinal cord, this work corroborates the previously introduced concept of pseudo-superdiffusion and underlines the potential of subdiffusion (α-metrics) to obtain local and complementary information compared to conventional DMRI techniques.

## Data Availability Statement

The original contributions presented in the study are included in the article/Supplementary Material, further inquiries can be directed to the corresponding author/s.

## Author Contributions

AC and SC: conceptualization. AC, GB, GTR, BA, AT, and SC: data curation. SC: funding acquisition. AC, BA, and SC: investigation. SC, AC, BA, and AT: methodology. AC, GB, and GTR: software. SC, AT, and FW: supervision. AC, GB, GTR, AT, BA, FW, and SC: validation. AC, GTR, SC, and BA: visualization. AC and SC: writing – original draft. AC, GB, GTR, AT, BA, FW, and SC: review and editing. All authors contributed to the article and approved the submitted version.

## Conflict of Interest

The authors declare that the research was conducted in the absence of any commercial or financial relationships that could be construed as a potential conflict of interest.

## Publisher’s Note

All claims expressed in this article are solely those of the authors and do not necessarily represent those of their affiliated organizations, or those of the publisher, the editors and the reviewers. Any product that may be evaluated in this article, or claim that may be made by its manufacturer, is not guaranteed or endorsed by the publisher.
