# Supplementary material for: Transient Anomalous Diffusion MRI in Excised Mouse Spinal Cord: Comparison Among Different Diffusion Metrics and Validation With Histology
Source: Front Neurosci. 2022 Feb 15;15:797642. doi: 10.3389/fnins.2021.797642 (PMC8885723; doi:10.3389/fnins.2021.797642)
Supplement: Supplementary file 1 [file Presentation_1.pdf]

Supplementary Material

1. Appendix 1: quantitative analysis of optical microscopies

The customized script to extract features from the optical microscopies of the mouse spinal cord was compiled in MATLAB (MATLAB R2016a, Natick, Massachusetts: The MathWorks Inc.), referring to the method adopted by Comin et al. [1]. The script takes in input the selected optical microscopies of each region of interest (ROI) placed in the white matter (WM) of mouse spinal cord and reads the information in terms of pixels intensity. Then the following steps are executed:

- image calibration, thus converting pixels into μm (the scale factor used is 1 pixel = 0.0333 μm) by measuring how many pixels are included in the unit bar in the image;
- low-pass filtering, using a pixel-wise adaptive Wiener method, based on statistics estimated from a local neighborhood of each pixel [2]. The size of the neighborhoods is provided as a σ value, and set as 6 by default, which in the case of Comin's paper [1] corresponds to σ = 0.025 µm, while in our case corresponds to a σ = 0.2 µm;
- segmentation of the image into three tissue classes, to compute the fractions of intra- and extra-cellular space (ics and ecs, respectively) and myelin (myel). A fourth class is considered, including all the elements not clearly attributable to the other three, with the normalization rule f_ics + f_ecs + f_myel + f_other = 1.

The script is set up to identify the objects given by the sum of ics-mask and other-mask, since these masks individuate the areas with the highest intensity, corresponding to the axolemma. Other structures non-attributable to axons are discarded in the following steps, through the application of geometric selection criteria.

1.1 Selection rules

The next step is the cropping of a homogeneous area of the ROI, in order to extract further histologic characteristics of white matter tracts, such as axon diameter, the standard deviation of the distribution of axon diameters, axonal density, and the effective local density.

Some selection rules are also described in the Methods section of Comin's paper [1]:

- surface threshold: by taking the ics mask as an input, the function allows the user to select the minimum axon diameter (minimum area), in order to set a cut-off on the object area, excluding thus speckles and non-axons spots with less pixels than the lower threshold;
- circularity threshold: the objects passing the first high-pass filter are detected through a Canny edge detector (with a threshold set to 0.99). Those objects with a ratio of perimeter to square root of area larger than 6.2 are discarded. This means that, approximating the axial section of axons to an ellipse, the cut-off on circularity translates into discarding objects with a ratio $\frac{P}{\sqrt{A}} = \frac{2\pi\cdot\sqrt{\frac{a^2+b^2}{2}}}{a\cdot b\cdot\pi} > 6.2$, which corresponds to all the elongated ellipses with b > 6a (where a and b are the semi-minor and semi-major axes of the ellipse, respectively);

- uniformity thresholds: pixels belonging to the same tissue compartment are likely to present homogeneous intensities. The objects with a ratio between the standard deviation and the mean intensity $\frac{\sigma}{\mu} > 0.5$ are discarded, as supposed to contain more than one tissue, or debris.

Furthermore, a visual check is performed, and objects erroneously attributed to axons are manually selected, and deleted.

1.2 Measurement of morphological parameters

All those objects passing the selection rules and the visual check are considered by another function, which extracts the morphological quantities of interest. Two types of diameters are considered:

- the Feret diameter, corresponding to the diameter of an isoperimetric circumference (with the same perimeter as the object);
- the diameter of an equivalent circle (with the same area as the object's one; see Ong et al.'s paper [3]).

Axons are polydisperse, therefore there is a distribution of axon sizes: we assumed this distribution to be a gaussian one. The mean axons diameters and the related standard deviations ($SD_{ax.diam}$) are thus derived from a gaussian fit, for each considered white matter region.

The axonal density is computed as the ratio between the number of objects (passing the selection rules) and the area of the cropped region in the white matter tract, measured in μm^2.

We also calculated an 'effective local density' (eld), considering the distribution of nearest neighbors' distances from a given object, to the n-th nearest neighbor. The eld is evaluated by fitting the power law behavior $\langle r_n \rangle \sim \sqrt{\frac{n}{\rho\pi}}$, which, for $n \geq 8$ provides values of effective local density with a relative error lower than 0.016, as described in Comin et al. [1]. The effective local density is an estimate of the local axonal density, ignoring the presence of large free-axons region (for example the blobs containing myelin and axons debris), or of neuron nuclei and blood vessels. The function calculates the effective local density from the intercept of the linear regression. A region with isolated, clustered, axon-free spots will provide an effective local density higher than the axon density, because the positions allowable for occupation are less, with a consequent increasing of the axon density; a region with many, homogeneously distributed axon-free spots, will provide a lower effective local density.

1.3 Optical microscopy and ROIs selection

The mouse spinal cord was cut at three different levels and analyzed with a Zeiss Axioskop light microscope. Optical images of the fixed mouse spinal cord were collected at the thoracic level (approximately at the level T3), thoracic-lumbar sections and at the lumbar enlargement, (approximately at the L7 level - **Supplementary Figure 1**). Specifically, several regions of interest (ROIs) were scanned at a higher magnification. Among the selected ROIs (**Supplementary Figure 2**), only one included ascending-sensory fibers (funiculus gracilis, *fg*), while the others were placed in descending-motor tracts: dorsal Cortico-Spinal Tract, Rubro-Spinal Tract, Reticulo-Spinal Tract, medial Vestibulo-Spinal Tract, Spino-Thalamic Tract (respectively, *dCST*, *RST*, *ReST*, *VST*, *STT*).
By using histological reference images, the ROIs were manually drawn by three different operators referring to the mouse white matter ATLAS, and subsequently intersected.

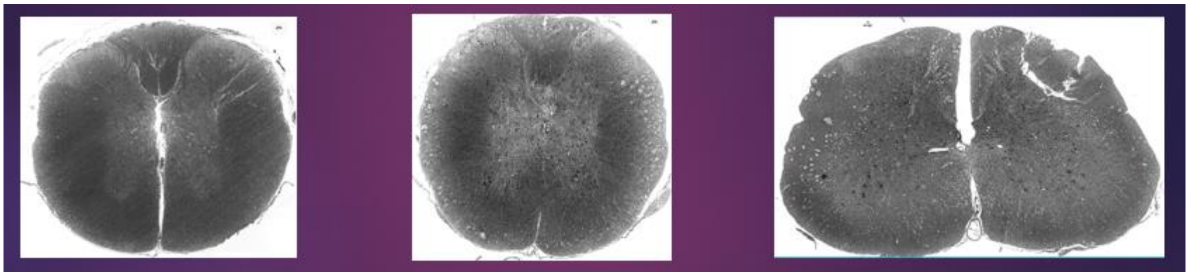

Supplementary Figure 1. The optical microscopy was performed at three levels. From left to right, the thoracic section, the thoracic-lumbar section, and the lumbar enlargement are shown. Each image corresponds to a magnification of x20. The tiny scalebar at the top corresponds to 20 μm.

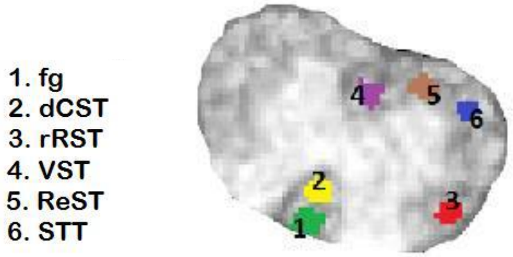

Supplementary Figure 2. The ROIs selected in the spinal cord white matter. The dorsal tracts are the funiculus gracilis, fg (1) and the dorsal Cortico-Spinal Tract, dCST (2); the lateral tract considered is the right Rubro-Spinal Tract, rRST (3); the ventral tracts are the Vestibulo-Spinal Tract, VST (4), the Reticulo-Spinal Tract, ReST (5), and the Spino-Thalamic Tract, STT (6). The regions of interest (ROIs) are superimposed on a Mγ map.

2. Appendix 2: Linear regression between γ-metrics, α-metrics and histologic features

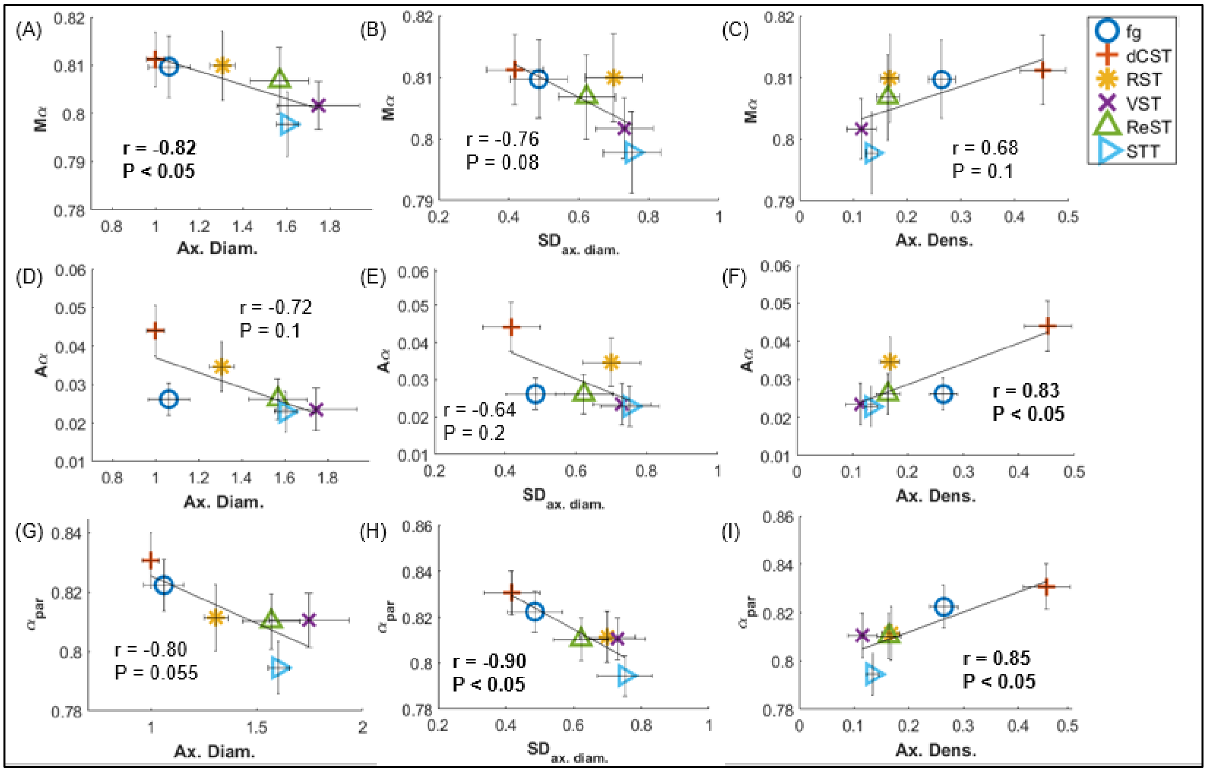

Supplementary Figure 3. Correlation plots between α-imaging metrics and histologic features. Pearson's correlation coefficient r and the significance level P are indicated in the plots. Fg = funiculus gracilis; dCST = dorsal Cortico-Spinal Tract; RST = rubro-spinal tract; VST = vestibulo-spinal tract; ReST = reticulo-spinal tract; STT = spino-thalamic tract; Ax. Diam. = axonal diameter; $SD_{ax.diam.}$ = standard deviation of the distribution of axonal sizes; Ax. Dens. = derived axonal density.

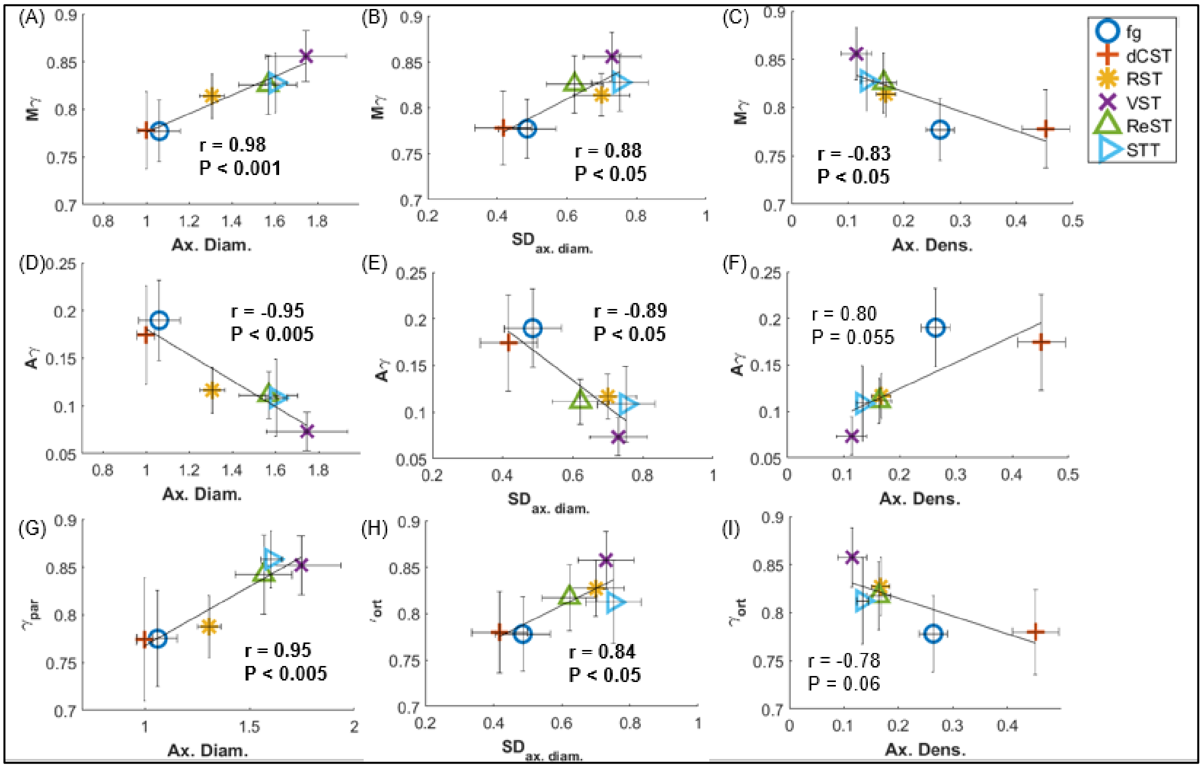

Supplementary Figure 4. Correlation plots between γ-imaging metrics and histologic features. Pearson's correlation coefficient r and the significance level P are indicated in the plots. Fg = funiculus gracilis; dCST = dorsal Cortico-Spinal Tract; RST = rubro-spinal tract; VST = vestibulo-spinal tract; ReST = reticulo-spinal tract; STT = spino-thalamic tract; Ax. Diam. = axonal diameter; $SD_{ax.diam.}$ = standard deviation of the distribution of axonal sizes; Ax. Dens. = derived axonal density.

3. Appendix 3: Agreement between the histologic features and literature

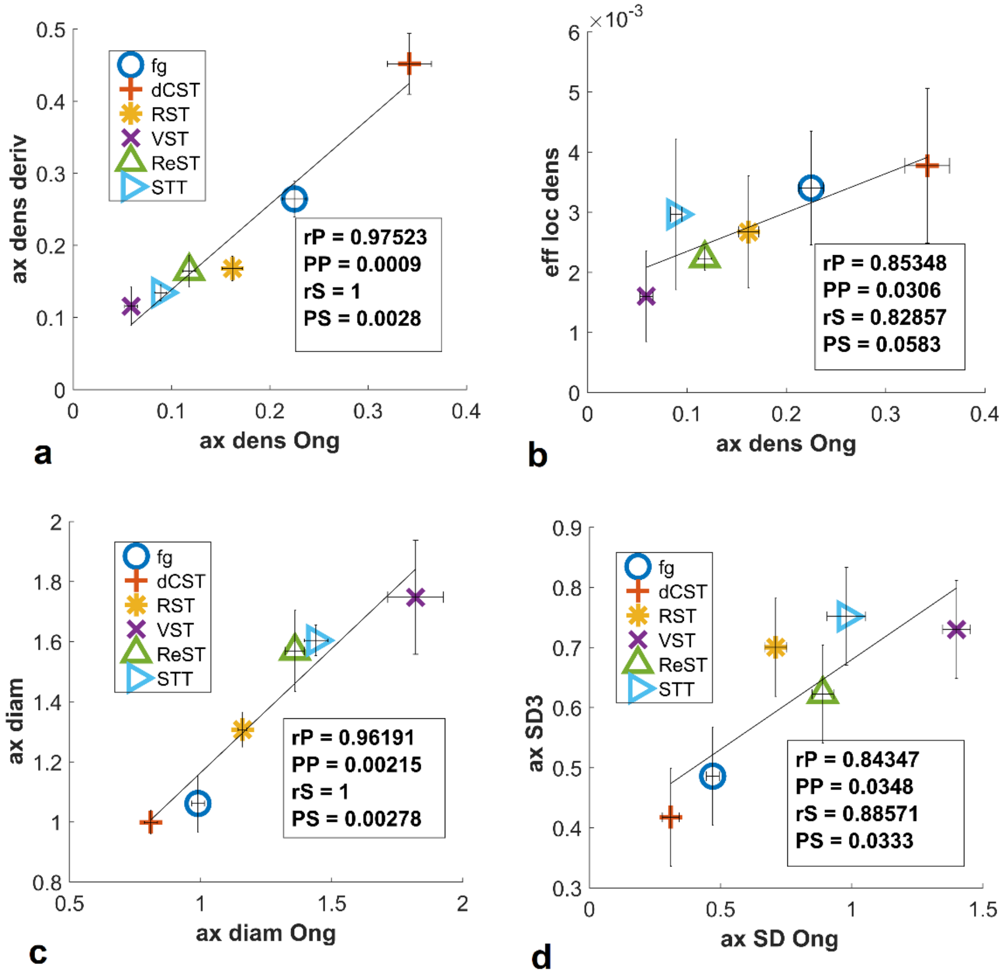

Supplementary Figure 5. Structural parameters derived from the processing of light microscopy images plotted against structural parameters derived in Ong et al. using TEM images. The derived axonal density and the effective local density are plotted against the axonal density computed in Ong et al.[3] in a) and b); the axon diameter (ax diam) and the standard deviation of the distribution of diameters (ax SD) are plotted against the analogous structural parameters in c) and d). rP=Pearson's correlation coefficient; PP=significance level; rS=Spearman's correlation coefficient; PS=significance level.

References

1. Comin CH, Santos JR, Corradini D, Morrison W, Curme C, Rosene DL, et al. Statistical physics approach to quantifying differences in myelinated nerve fibers. Scientific reports 2014; 4.
2. Lim JS. Two-dimensional signal and image processing. Englewood Cliffs, NJ, Prentice Hall, 1990, 710 p. 1990.
3. Ong HH, Wright AC, Wehrli SL, Souza A, Schwartz ED, Hwang SN, et al. Indirect measurement of regional axon diameter in excised mouse spinal cord with q-space imaging: simulation and experimental studies. Neuroimage 2008; 40: 1619-1632.
